# Nutrient addition shifts plant community composition towards earlier flowering species in some prairie ecoregions in the U.S. Central Plains

**DOI:** 10.1371/journal.pone.0178440

**Published:** 2017-05-26

**Authors:** Lori Biederman, Brent Mortensen, Philip Fay, Nicole Hagenah, Johannes Knops, Kimberly La Pierre, Ramesh Laungani, Eric Lind, Rebecca McCulley, Sally Power, Eric Seabloom, Pedro Tognetti

**Affiliations:** 1 Department of Ecology, Evolution and Organismal Biology, Iowa State University, Ames, Iowa, United States of America; 2 USDA-ARS Grassland Soil and Water Research Lab, United States Department of Agriculture–Agricultural Research Service, Temple, Texas, United States of America; 3 School of Life Sciences, University of KwaZulu-Natal, Pietermaritzburg, South Africa; 4 School of Biological Science, University of Nebraska, Lincoln, Nebraska, United States of America; 5 Department of Integrative Biology, University of California, Berkeley, California, United States of America; 6 Department of Biology, Doane University, Crete, Nebraska, United States of America; 7 Department of Ecology, Evolution, and Behavior, University of Minnesota, St. Paul, Minnesota, United States of America; 8 Department of Plant and Soil Sciences, University of Kentucky, Lexington, Kentucky, United States of America; 9 Hawkesbury Institute for the Environment, Western Sydney University, Richmond, New South Wales, Australia; 10 IFEVA, Universidad de Buenos Aires, CONICET, Facultad de Agronomía, Buenos Aires, Argentina; Universite du Quebec a Chicoutimi, CANADA

## Abstract

The distribution of flowering across the growing season is governed by each species’ evolutionary history and climatic variability. However, global change factors, such as eutrophication and invasion, can alter plant community composition and thus change the distribution of flowering across the growing season. We examined three ecoregions (tall-, mixed, and short-grass prairie) across the U.S. Central Plains to determine how nutrient (nitrogen (N), phosphorus, and potassium (+micronutrient)) addition alters the temporal patterns of plant flowering traits. We calculated total community flowering potential (FP) by distributing peak-season plant cover values across the growing season, allocating each species’ cover to only those months in which it typically flowers. We also generated separate FP profiles for exotic and native species and functional group. We compared the ability of the added nutrients to shift the distribution of these FP profiles (total and sub-groups) across the growing season. In all ecoregions, N increased the relative cover of both exotic species and C_3_ graminoids that flower in May through August. The cover of C_4_ graminoids decreased with added N, but the response varied by ecoregion and month. However, these functional changes only aggregated to shift the entire community’s FP profile in the tall-grass prairie, where the relative cover of plants expected to flower in May and June increased and those that flower in September and October decreased with added N. The relatively low native cover in May and June may leave this ecoregion vulnerable to disturbance-induced invasion by exotic species that occupy this temporal niche. There was no change in the FP profile of the mixed and short-grass prairies with N addition as increased abundance of exotic species and C_3_ graminoids replaced other species that flower at the same time. In these communities a disturbance other than nutrient addition may be required to disrupt phenological patterns.

## Introduction

Assembly rules suggest that communities assemble to limit interspecific niche overlap, such that the exploitation of shared resources is minimized and resource use by the community as a whole is relatively efficient [1, 2]. One means of limiting interspecific niche overlap is to separate the onset and length of life history events along a temporal (phenological) gradient [3]. For example, plant community assembly tends to reduce interspecific overlap in the phenology of flower production, which limits pre-dispersal seed predation and competition for pollinator visits [4, 5]. Other organisms, such as mycorrhizal fungi [6] and higher trophic groups [7], also benefit from temporal dispersion of plant production. Furthermore, the continuity of ecosystem functions, such as water and nutrient uptake and carbon fixation, also rely on the phenological sequence of plant growth and reproduction across the growing season [8].

Flowering phenology is constrained by evolutionary processes [9] and each species has its own characteristic set of triggers that initiate life history events, such as reproduction [10]. Climate conditions, such as water availability and accumulated growing degree days, trigger flowering in some species, and thus their phenology can vary from year to year in response to inter-annual climate variability [11]. In contrast, some phenological events are initiated by events, such as day length, that are fixed in time [12]. At the community level, however, the sequence of flowering across species is relatively consistent from year to year [13, 14]. Therefore, we can use gross estimates of phenological activity to develop profiles of community-level phenological activity.

Compositional shifts concomitant with patterns in global change such as eutrophication and invasion by exotic species have consequences for the timing and distribution of phenological activity at the community scale. Plant communities are exposed to high levels of nutrient input [15]. Indeed, eutrophication is disrupting worldwide grasslands [16] by reducing plant diversity and community function [17, 18, 19] and increasing exotic species cover and richness [20]. Here, we compare two competing hypotheses concerning intra-community phenological patterns, niche collapse and sparse-niche exploitation, that could follow these community changes. Under the niche collapse scenario (Hypothesis 1) the nutrient-induced loss of native species diversity may push novel communities towards dominance by a few species that share life-history strategies [21], including the onset and length of flowering [22]. This scenario would result in a collapse of the community’s reproductive activity into a narrower window.

In contrast to narrowing the phenology of flowering, nutrient addition could shift, or broaden, the distribution of flowering activity to sparsely occupied temporal niches (Hypothesis 2). This could occur if nutrients facilitate the immigration or an increase in species that exploit an underutilized resource, such as a window of time when few established species are flowering [23]. These sparsely-occupied phenological windows can occur at any point in a growing season, but are typically in the shoulder seasons, which are early spring and late fall in the U.S. Central Plains [24]. Such phenological mismatches between increasing and established species reduce barriers to invasion, facilitating, and possibly accelerating, plant species turnover [25].

We use data from a standardized, distributed experiment across three ecoregions of the U.S. Central Plains to test these two contrasting hypotheses following nutrient [N, P, K (+micronutrients)] addition. These prairie ecoregions (tall-, mixed-, and short-grass) are primarily differentiated by climate, specifically mean annual precipitation. As an estimate of plant phenological response, we developed the flowering potential (FP) metric to evaluate changes in community-scale phenological traits. To better evaluate those factors underlying change in the total community we further sub-divided these data into provenance and functional groups. Finally, we compared the volume of the total community FP profiles to determine if the overall phenological window is narrowing as a result of niche collapse (additional support for Hypothesis 1).

## Materials and methods

### Nutrient treatment and site selection

Plant cover data were collected as part of the Nutrient Network (nutnet.org) [18, 19, 26]. In this experiment, plots are 5 x 5 m and arranged in blocks. Sites typically have three blocks, but some have as many as six. Three nutrient treatments [N, P and K (+ micronutrients, hereafter “μ”)], each with two levels (control, added), are crossed in a factorial design, for a total of eight treatment combinations per block. Nutrient addition rates and sources are: 10 g m^−2^ year^−1^ N, as timed-release urea [(NH_2_)_2_CO], 10 g m^−2^ year^−1^ P, as triple-super phosphate [Ca(H2PO_4_)_2_], 10 g m^−2^ year^−1^ K, as potassium sulfate [K_2_SO_4_], and 100 g m^−2^ of a micronutrient mix of Fe (15%), S (14%), Mg (1·5%), Mn (2·5%), Cu (1%), Zn (1%), B (0·2%) and Mo (0·05%). N, P and K are applied annually, micronutrients (+μ) were applied only once at the start of the experiment to avoid toxicity. Plant species cover is estimated to the nearest percent at peak biomass using a modified Daubenmire [27] method in permanent 1 x 1 m subplots. Because plant canopies can overlap, total cover data can sum to greater than 100% and we converted this data to relative cover by dividing each individual species’ cover by the plot total. Plant relative cover data are available on Figshare [28].

To limit variation due to rainfall seasonality and growing season length, we restricted our analysis to U.S. Central Plains grassland ecoregions that were identified by local researchers as tall-, mixed-, or short-grass prairie. We chose to use data from the fourth treatment year, as community composition shifts significantly over the first 3 years of nutrient application [18]. This resulted in a total of 11 sites (Table 1).

**Table 1:** Nutrient Network sites used in this analysis.

Site	Ecoregion	U.S. State	Latitude (°N)	Longitude (°W)	Year 4	MAT (°C)	MAP (mm)
**Boulder South Campus**	Short-	Colorado	39.97	-105.23	2012	9.7	425
**Shortgrass Steppe LTER**	Short-	Colorado	40.82	-104.77	2011	8.4	365
**Cedar Point**	Short-	Nebraska	41.20	-101.63	2011	9.5	445
**Bart's Brothers**	Mixed-	Nebraska	42.24	-99.65	2011	8.7	597
**Saline Experimental Range**	Mixed-	Kansas	39.05	-99.10	2011	11.8	607
**Temple**	Tall-	Texas	31.04	-97.35	2011	19.1	871
**Konza LTER**	Tall-	Kansas	39.07	-96.58	2011	11.9	877
**Chichaqua Bottoms Greenbelt**	Tall-	Iowa	41.79	-93.39	2013	9.0	855
**Cedar Creek LTER**	Tall-	Minnesota	42.43	-93.21	2011	6.3	750
**Trelease**	Tall-	Illinois	40.08	-88.83	2012	11.0	982
**Hall's Prairie**	Tall-	Kentucky	36.87	-86.70	2011	13.6	1282

The Nutrient Network North American prairie sites used in this analysis with site ecoregion, U.S. state, latitude and longitude, the year the fourth fertilization treatment occurred, mean annual temperature (MAT), and mean annual precipitation (MAP).

See nutnet.org for site contact information.

### Plant phenology and species characteristics

The FP profile uses published flowering intervals to distribute peak-season plant cover values across the growing season, allocating each species’ cover value to only those months in which it typically flowers. To create this metric, we recorded the month(s) each species typically flowers from published floras [29, 30]. These data were primarily obtained from the *Flora of the Great Plains* [31], but for species not found there we used other floras [32, 33, 34, 35]. For example, *Monarda Fistulosa* L. flowers in June, July, and August. For each month the relative cover of each species at peak biomass in each plot was multiplied by a weight of 1, if the flora indicated it was expected to flower, or by 0 if it was not expected to flower in that month. These weighted relative cover values were then summed for all species in each plot for that month. To continue the example, *M*. *fistulosa*’s relative cover is 0.117 for plot 61 at Chicaqua Bottoms green belt, and is added to the FP value for the months June, July, and August for that plot. Species that do not flower in a given month thus do not contribute to its FP value. In our example, *M*. *fisulosa*’s cover would not added to the FP profile of May. The complete dataset of plant traits, including species, reported flowering month(s), functional group, and consulted flora, are available on Figshare [36].

We determined species origin from the United States Department of Agriculture’s Plants database (plants.usda.gov). Osborne et al. [37] was consulted to determine the photosynthetic pathway for each graminoid species. Data excluded from the analysis include plants that were only identified to genus (e.g. *Carex* sp.) and tree seedlings. To determine the influence of species’ provenance or functional group on the total FP we used the above method on subsets of the data. This generated FP profiles for exotic and native species of all functional types (provenance), and also C_3_ graminoids, C_4_ graminoids, forbs, and legumes of all origins (functional group).

### Analysis

All analyses were conducted using R version 3.3.0 [38]. The FP profiles (total and the exotic, native, forb, C_3_ and C_4_ graminoid, and legume sub-sets) were analyzed with a linear mixed model (nlme package) [39] with nutrients (N, P, and K+μ), growing season month (May through October), and ecoregion (tall-, mixed-, or short-grass), and their interactions as the model’s fixed effects and block nested within site included as a random effect. We used Bonferroni multiple comparison corrections (lsmeans function) [40] to determine the significance of the nutrient treatments, month, and ecoregion on the FP profile. As the main effects of the fertilizer treatments on plant productivity and community composition are discussed elsewhere [18, 19], we emphasize those results where the nutrient treatments significantly interact with month, because these interactions indicate a change in the distribution of reproduction across the growing season. Nutrient effects and interactions between month and ecoregion are reported, but interpretation of these effects is outside the scope of this study.

We also calculated the total community flowering volume (area under the curve) generated by the total community data using the auc function in the flux package [41]. The nutrient effect on curve volume (January through December) was analyzed with a linear mixed model with nutrients (N, P, and K+μ), ecoregion (tall-, mixed-, or short-grass), and their interactions as fixed effects and block nested within site included as a random effect.

## Results

**Fig 1:**
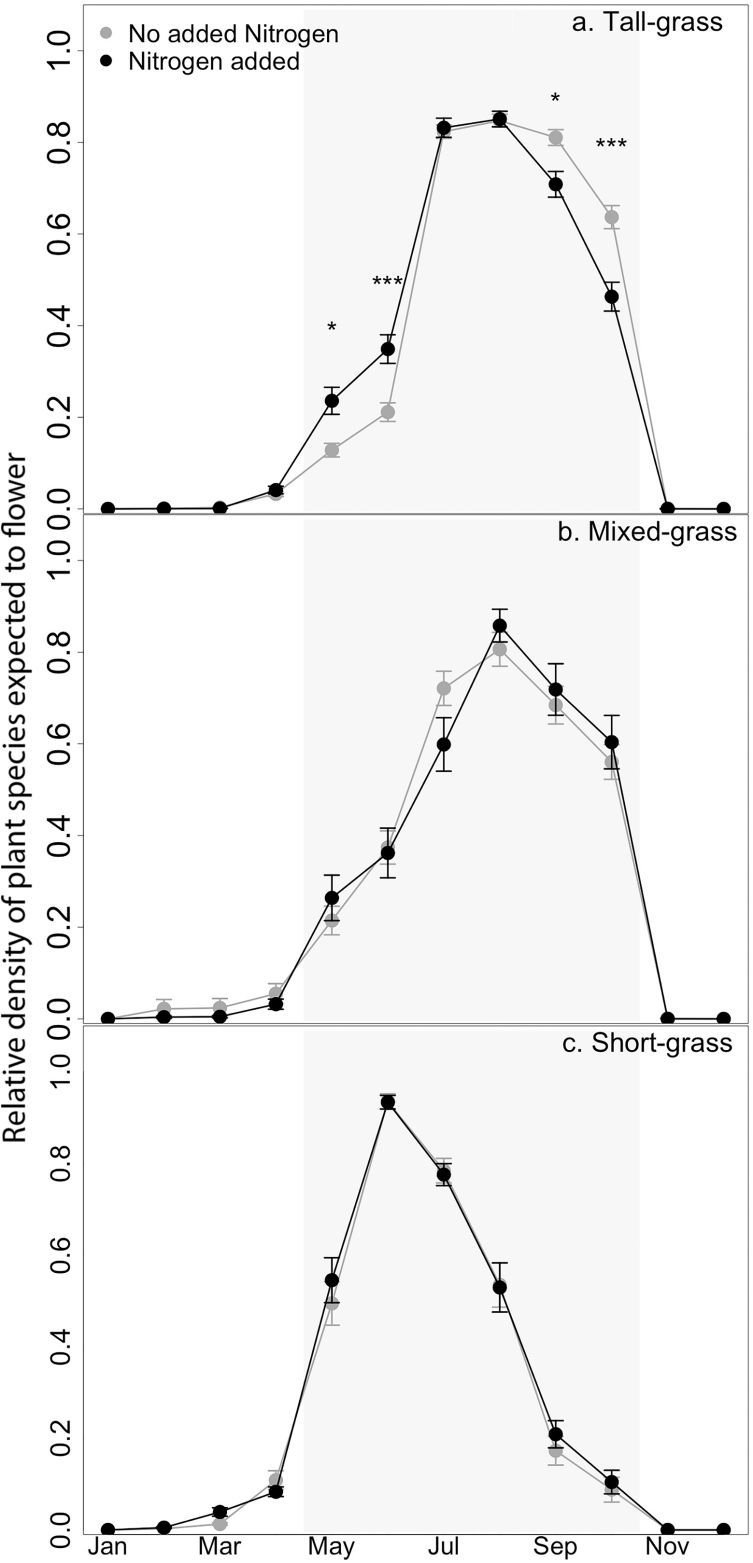
Total flowering potential for prairie ecoregions. Mean (± SE) total flowering potential (FP), or the relative cover of species expected to flower in each month, in tall- (top), mixed- (middle), and short-grass (bottom) prairies. FP values for the entire year illustrate the total community flowering volume (area under the curve); the shaded ecoregion indicates the growing season in the U.S. central plains. Treatments without added nitrogen (N) include the control, phosphorus (P), potassium + micronutrients (K+μ), and PK+μ, and those with added N include N, NP, NK+μ, and NPK+μ. Asterisks indicate values that were determined to be statistically distinct following Bonferroni correction (*** p < 0.001, ** p < 0.01, *p < 0.05).

We found that N addition shifted the distribution of the total FP profile across months, but this differed among the studied ecoregions (Table 2; S1 Table). Bonferroni post-hoc tests revealed that N addition influenced the FP in the tall-grass ecoregion, where the relative cover of species that typically flower in May and June increased in plots with added N (i.e. N, P*N, K*N, and P*K+μ*N) compared with plots with no added N (i.e. control, P, K+μ, and P*K+μ) (Fig 1A). Furthermore, those species that typically flower in September and October decreased with added N. In contrast to the tall-grass ecoregion, added N did not change the total FP profiles in the mixed- (Fig 1B), and short-grass prairie (Fig 1C). The overall flowering volume (area under curve; 307.0 ± 3.0% cover, mean ± standard error) was not affected by the nutrient treatments or ecoregion (Fig 1; Table 3).

**Table 2:** ANOVA table for the flowering potential analyses of the total community, exotic species, and native species.

		Total FP			Exotic species FP			Native species FP		
	n.d.f.	d.d.f	F-value		d.d.f	F-value		d.d.f	F-value	
**Intercept**	1	1751	885.71	***	1741	8.48	*	1739	183.18	***
**Phosphorus (P)**	1	1751	0.03		1741	3.14		1739	1.57	
**Potassium + micros (K+μ)**	1	1751	0.01		1741	0.57		1739	0.25	
**Nitrogen (N)**	1	1751	0.11		1741	59.26	***	1739	24.65	***
**Month**	5	1751	254.37	***	1741	19.45	***	1739	275.05	***
**Ecotype**	2	8	0.48		8	0.24		8	0.49	
P * K+μ	1	1751	1.26		1741	0.02		1739	0.85	
P * N	1	1751	0.03		1741	0.46		1739	0.47	
K+μ * N	1	1751	0.09		1741	1.28		1739	0.27	
P * Month	5	1751	0.37		1741	0.43		1739	0.23	
K+μ * Month	5	1751	0.41		1741	0.10		1739	0.67	
N * Month	5	1751	7.53	***	1741	2.92	*	1739	2.91	*
P * Ecotype	2	1751	0.17		1741	10.05	***	1739	5.74	**
K+μ * Ecotype	2	1751	0.08		1741	0.25		1739	0.11	
N * Ecotype	2	1751	0.30		1741	0.26		1739	0.55	
Month * Ecotype	10	1751	146.76	***	1741	5.39	***	1739	127.68	***
P * K+μ * N	1	1751	0.16		1741	1.02		1739	0.57	
P * K+μ * Month	5	1751	0.25		1741	0.17		1739	0.40	
P * N * Month	5	1751	0.24		1741	0.10		1739	0.16	
K+μ * N * Month	5	1751	0.23		1741	0.12		1739	0.11	
P * K+μ * Ecotype	2	1751	0.02		1741	0.18		1739	0.23	
P * N * Ecotype	2	1751	0.26		1741	2.01		1739	2.50	
K+μ * N * Ecotype	2	1751	0.01		1741	2.61		1739	1.29	
P * Month * Ecotype	10	1751	0.51		1741	0.38		1739	0.48	
K+μ * Month * Ecotype	10	1751	0.78		1741	1.00		1739	0.70	
N * Month * Ecotype	10	1751	3.61	***	1741	0.93		1739	2.48	**
P * K+μ * N * Month	5	1751	0.15		1741	0.22		1739	0.01	
P * K+μ * N * Ecotype	2	1751	0.47		1741	6.61	**	1739	2.45	
P * K+μ * Month * Ecotype	10	1751	0.49		1741	0.10		1739	0.43	
P * N * Month * Ecotype	10	1751	0.21		1741	0.41		1739	0.20	
K+μ * N * Month * Ecotype	10	1751	0.42		1741	0.21		1739	0.34	
P * K+μ * N * Month * Ecotype	10	1751	0.29		1741	0.36		1739	0.22	

ANOVA table for flowering potential (FP) for the total community and the exotic and native components of the prairie communities, including the associated numerator degrees of freedom (n.d.f.) and denominator degrees of freedom (d.d.f.), F-values, and the associated p-values

*** p < 0.001

** p < 0.01

*p < 0.05.

**Table 3:** ANOVA table for area under the curve analysis.

	n.d.f.	d.d.f	F-value	
**Intercept**	1	261	706.97	***
**Phosphorus (P)**	1	261	0.03	
**Potassium (K+μ)**	1	261	0.03	
**Nitrogen (N)**	1	261	1.05	
**Ecotype**	2	8	0.08	
P * K+μ	1	261	2.17	
P * N	1	261	0.03	
K+μ * N	1	261	0.15	
P * Ecotype	2	261	0.07	
K+μ * Ecotype	2	261	0.03	
N * Ecotype	2	261	0.39	
P * K+μ * N	1	261	0.03	
P * K+μ * Ecotype	2	261	0.15	
P * N * Ecotype	2	261	0.28	
K+μ * N * Ecotype	2	261	0.05	
P * K+μ * N * Ecotype	2	261	0.10	

ANOVA table for the analyses of the area under the curve for the total community flowering potential (FP) Columns include the numerator degrees of freedom (n.d.f.) and denominator degrees of freedom (d.d.f.), F-values, and the associated p-values

*** p < 0.001.

**Fig 2:**
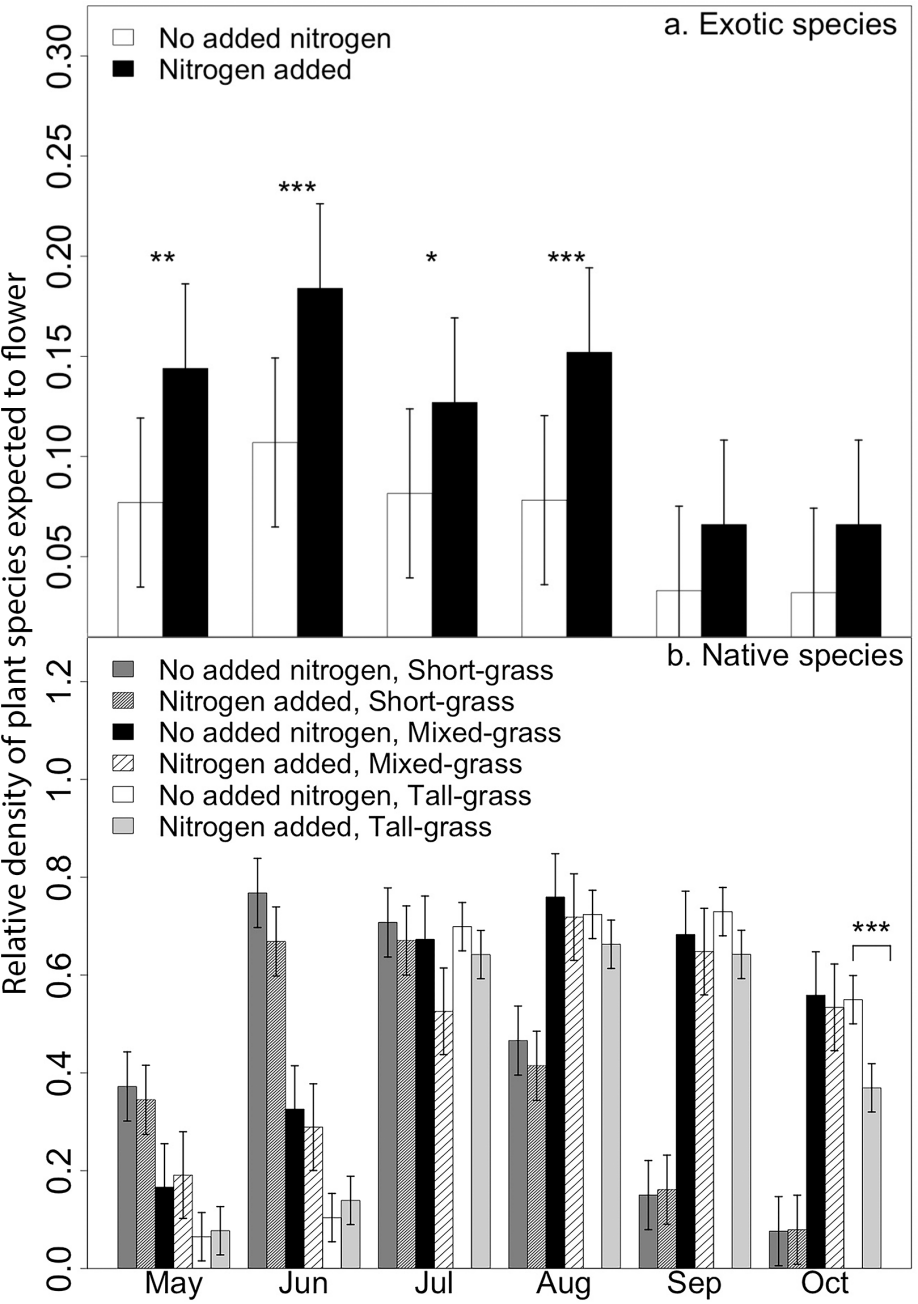
Flowering potential for exotic and native species. Mean (± SE) flowering potential (FP), the relative cover of species expected to flower in each month of the growing season. Exotic species FP (top) is averaged across all prairie ecoregions, as the highest order interaction was nitrogen (N) * month. Native species FP (bottom) is separated for the tall-, mixed-, and short-grass prairies because there was an N * month * ecoregion interaction for this group. Asterisks indicate values that are statistically distinct following Bonferroni correction *** p < 0.001, ** p < 0.01, *p < 0.05.

The FP profile for exotic species shifted in all communities following N addition (Table 2). The relative cover of exotic species typically flowering in May through August increased in those plots with added N compared with those treatments without N (Fig 2A). N addition changed the FP profile for native species (Table 2) and post-hoc tests indicate that N reduced in the cover of native species that typically flower in October, but only in the tall-grass ecoregion (Fig 2B).

**Fig 3:**
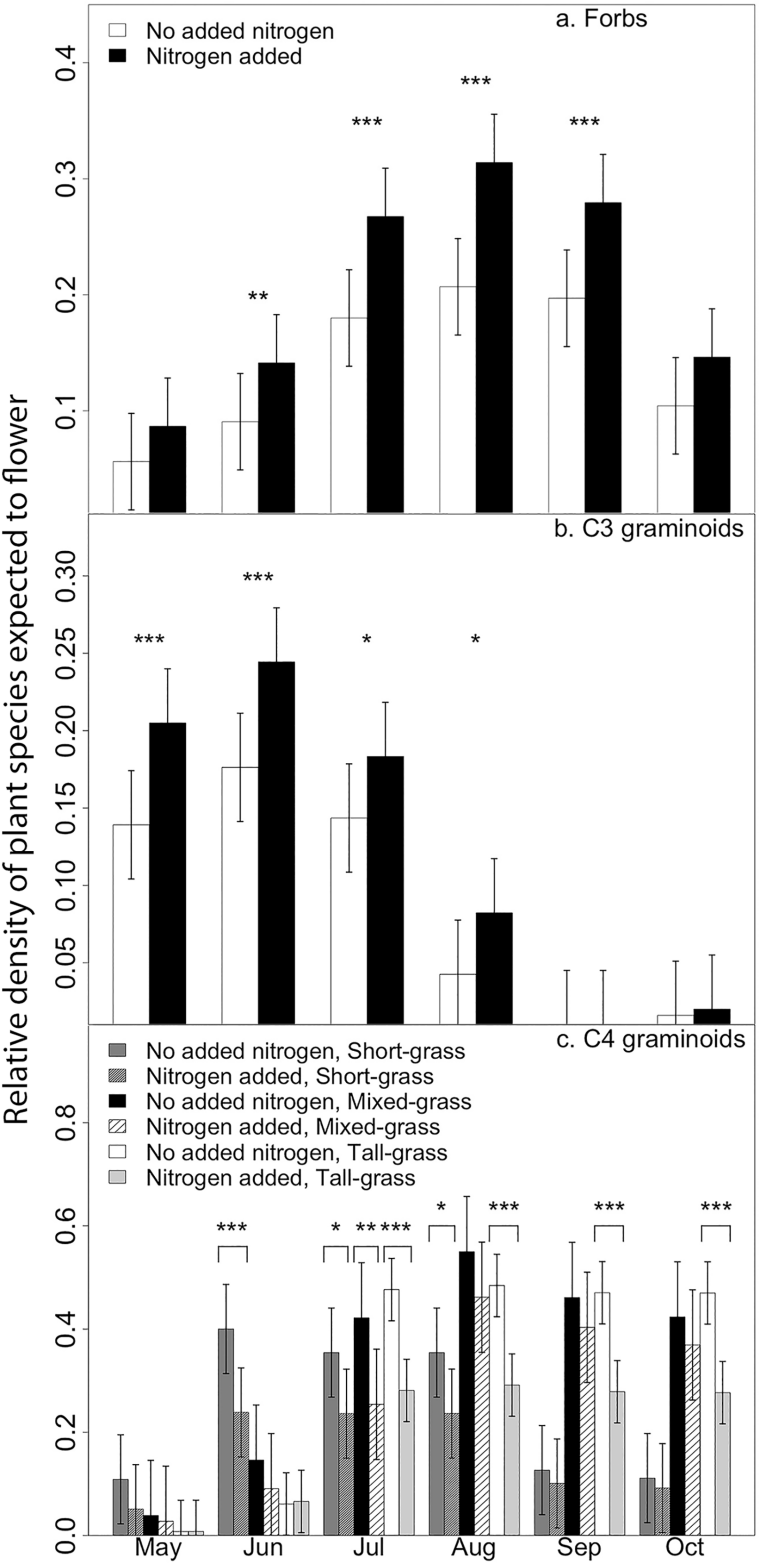
Flowering potential for plant functional groups. Mean (± SE) flowering potential (FP), the relative cover of species expected to flower in each month of the prairie growing season. FP for forb (top) and C_3_ graminoid (middle) functional groups are averaged across all prairie ecoregions, as the highest order interaction for these was nitrogen (N) * month. C_4_ graminoid FP (bottom) is provided for the tall-, mixed-, and short-grass prairies as there was an N * month * ecoregion interaction for this group. Asterisks indicate values that are statistically distinct *** p < 0.001, ** p < 0.01, *p < 0.05 (Bonferroni corrected).

The FP distribution across the growing season for both forbs and C_3_ graminoids changed following the addition of N compared to plots with no added N in all ecoregions (Table 4). Post-hoc tests find that N addition increased the relative cover of forbs that typically flower in June through September (Fig 3A). The relative cover of C_3_ graminoids that typically flower from May through August also increased with N addition (Fig 3B).

**Table 4:** ANOVA table for flowering potential analysis for plant functional groups.

		Forb FP			C_3_ gramminoid FP			C_4_ gramminoid FP			Legume FP		
	n.d.f.	d.d.f	F-value		d.d.f	F-value		d.d.f	F-value		d.d.f	F-value	
**Intercept**	1	1751	30.12	***	1753	9.02	*	1763	34.93	***	1759	5.55	*
**Phosphorus (P)**	1	1751	0.12		1753	4.06	*	1763	2.41		1759	0.02	
**Potassium + micros (K+μ)**	1	1751	0.02		1753	0.37		1763	1.39		1759	11.43	***
**Nitrogen (N)**	1	1751	80.99	***	1753	48.08	***	1763	187.23	***	1759	38.96	***
**Month**	5	1751	108.64	***	1753	107.07	***	1763	198.48	***	1759	4.46	***
**Ecotype**	2	8	1.03		8	1.95		8	0.35		8	2.06	
P * K+μ	1	1751	0.31		1753	0.10		1763	4.86	*	1759	5.04	*
P * N	1	1751	1.53		1753	2.12		1763	0.09		1759	0.62	
K+μ * N	1	1751	0.24		1753	1.47		1763	0.87		1759	0.86	
P * Month	5	1751	0.56		1753	0.89		1763	0.14		1759	0.06	
K+μ * Month	5	1751	0.06		1753	0.21		1763	0.32		1759	0.38	
N * Month	5	1751	4.57	***	1753	3.25	**	1763	9.88	***	1759	0.52	
P * Ecotype	2	1751	1.43		1753	5.91	**	1763	1.96		1759	0.81	
K+μ * Ecotype	2	1751	0.40		1753	0.31		1763	1.42		1759	10.93	***
N * Ecotype	2	1751	1.19		1753	2.15		1763	4.71	**	1759	3.01	*
Month * Ecotype	10	1751	21.13	***	1753	40.32	***	1763	45.69	***	1759	5.58	***
P * K+μ * N	1	1751	0.00		1753	0.38		1763	0.46		1759	5.99	*
P * K+μ * Month	5	1751	0.16		1753	0.11		1763	0.63		1759	0.23	
P * N * Month	5	1751	0.11		1753	0.47		1763	0.03		1759	0.15	
K+μ * N * Month	5	1751	0.34		1753	0.17		1763	0.26		1759	0.34	
P * K+μ * Ecotype	2	1751	1.13		1753	0.20		1763	1.60		1759	1.88	
P * N * Ecotype	2	1751	0.07		1753	0.73		1763	1.35		1759	10.75	***
K+μ * N * Ecotype	2	1751	2.05		1753	1.21		1763	0.99		1759	0.19	
P * Month * Ecotype	10	1751	0.81		1753	0.81		1763	0.27		1759	0.09	
K+μ * Month * Ecotype	10	1751	0.39		1753	0.33		1763	0.84		1759	0.66	
N * Month * Ecotype	10	1751	0.98		1753	0.41		1763	4.77	***	1759	0.79	
P * K+μ * N * Month	5	1751	0.11		1753	0.14		1763	0.09		1759	0.07	
P * K+μ * N * Ecotype	2	1751	0.42		1753	2.19		1763	0.19		1759	1.70	
P * K+μ * Month * Ecotype	10	1751	0.38		1753	0.19		1763	1.29		1759	0.59	
P * N * Month * Ecotype	10	1751	0.16		1753	0.16		1763	0.08		1759	0.61	
K+μ * N * Month * Ecotype	10	1751	0.31		1753	0.27		1763	0.28		1759	0.40	
P * K+μ * N * Month * Ecotype	10	1751	0.16		1753	0.31		1763	0.02		1759	0.11	

ANOVA table for the analyses of the flowering potential (FP) profil≥e for the forb, C_3_ graminoid, C_4_ graminoid, and legume functional groups in the prairie communities. Columns include the numerator degrees of freedom (n.d.f.) and denominator degrees of freedom (d.d.f.), F-values, and Bonferroni corrected p-values

*** p < 0.001

** p < 0.01

*p < 0.05.

The FP profile for C_4_ graminoids decreased following N addition in all ecoregions, though the response in each ecoregion varied by month (Table 4). N addition significantly reduced the cover of C_4_ graminoids that typically flower in June through August in the short-grass prairie, July for the mixed-grass, and July through October in the tall-grass prairies (Fig 3C). There was no interaction between applied nutrients and flowering month for legumes.

## Discussion

The prairie ecosystems historically covered the U.S. Central Plains and were dominated of C_4_ graminoids and forbs. These communities produced soils rich in organic matter that in many instances have subsequently been used for agriculture. For example, less than one percent of the tall-grass prairie remains [42]. Those remnants that remain are subject to multiple pressures, including species loss, exotic species invasion, and altered disturbance regimes [17]. In our analysis, N was the only added nutrient that interacted with month (and ecoregion in some cases) to change the FP profiles. Other nutrients, alone or in an interaction with ecoregion, did change aspects of community composition, but because these interactions did not include month, the nutrient-driven changes in composition did not alter the distribution of flowering across the growing season and are, therefore, outside the scope of this study [18, 19].

N addition shifted the total community FP profile of the tall-grass prairie by increasing the cover of species that typically flower in May and June and reducing the cover of those species that typically flower in September and October. This result is consistent with Hypothesis 2 that nutrients are disrupting community structure and facilitating an increase in species in an under-used phenological window. This change is largely driven by an increase of exotic species and C_3_ graminoids that flower early in the growing season with a concomitant reduction of late-flowering native species and C_4_ graminoids [43]. This general pattern has been found in other studies of nutrient enrichment; C_3_ grasses often increase with N addition while the more nutrient-efficient perennial C_4_ graminoids typically lose dominance because access to space and light are preempted [21, 44]. These phenological shifts, which are the result of plant community compositional change, may be exacerbated by physiological responses to climate change. Munson and Long [45] examined herbarium data and found that over an interval of 118 years C_3_ grasses have accelerated flowering time while C_4_ grasses have delayed flowering to later in the season. This divergence in flowering time was also greater in more mesic environments.

By placing these compositional shifts in a phenological framework we can appreciate the additional consequences for the community-level expression of flowering and reproduction, which, in turn, affect resource availability for higher trophic levels (e.g., herbivores, pollinators) and ecosystem function [5, 25]. Plants that flower early in the season also begin physiological activity earlier and this increases early demand for nutrients [8] and water, which can reduce soil storage and amplify drought conditions in subsequent months [46]. The timing and intensity of disturbances, such as fire, may also be affected because the presence and amount of senesced biomass shifts in time [47]. The N-induced decrease in plant activity at the end of the tall-grass growing season can also limit continuity of ecosystem function [48]. C_4_ graminoids are significant contributors to prairie production [49], nutrient cycling [50], and mutualistic interactions [9]. If the resources associated with species that are active late in the growing season are reduced, those organisms that rely on them can have difficulty provisioning for winter [5].

The tall-grass prairie ecoregion may be particularly vulnerable to spring-ward shifts in FP following a disturbance, such as eutrophication. The minimal cover of flowering species early in the growing season (May and June) provides a window of opportunity for early-flowering (and thus early growing) species to increase [4, 23]. It is unclear, however, if this sparse early-season niche is an inherent characteristic of the community [13], or an artifact of modern prairie management and restoration techniques [51].

In contrast, the early season increases in the cover of C_3_ graminoid and exotic species in the short- and mixed-grass ecoregions did not change the total community FP profile. Instead, these increasing groups replaced other spring-flowering species, particularly native species and C_4_ graminoids. These ecoregions may be more resistant to nutrient-driven early season phenological expansion because conditions in earlier months are too harsh for the present species pool [52]. In contrast, the low cover of short-grass species that flower in the late-season represents a potentially vulnerable phenological window for species invasion if the proper species pool and conditions align [25].

The mixed- and short-grass ecoregions may also be buffered from nutrient-induced changes in the total community FP profile because another resource, such as moisture or nocturnal temperature, is more limiting [11, 53]. Therefore, a perturbation of this (unidentified) limiting resource, in the right season, would be needed to induce phenological shifts in these plant communities [11, 14, 22].

This analysis is a conservative estimate of phenological change at the community level, as the published flowering intervals are broad temporally (monthly) and geographically and we use peak season (August/September) cover to estimate the FP profiles. As some early season species have senesced by this time, their cover estimates may be low. Therefore, our results that find treatment-induced increases in the cover of early season species may underestimate actual change. Actual observation of phenological patterns through high frequency sampling in the experiments will be necessary to resolve these effects, though the conservative estimates presented here do suggest an important phenological response to eutrophication.

## Conclusions

We found that N addition significantly altered the pattern of flowering across the season for the tall-grass prairie; specifically there was an increase in the relative cover of species that produce flowers early in the growing season (May and June) and a decrease in species that typically flower late in the growing season (September and October). This change supports our second hypothesis, that disturbance shifts the flowering activity by facilitating an increase of species that flower in otherwise sparsely occupied temporal niches. The spring-ward shift in potential phenological activity in the tall-grass ecoregion was driven by the increased abundance of exotic species and early flowering C_3_ graminoids. Declines in the cover of native species and C_4_ graminoids that flower late in the season were concomitant with the early-season changes. This temporal shift has consequences for ecosystem services and interactions with co-occurring species.

In contrast, we found no community-level phenological change occurring in either the mixed- and short-grass prairie ecoregions. These communities may be buffered from this type of change because the phenology of their potential invaders does not align with sparsely occupied phenological windows. Instead, invading species are replacing other species in an already occupied temporal niche. Our trait-based approach suggests that N addition can be a driver of phenological change in some communities. However, fine-grain sampling may reveal additional, subtler phenological change in response to eutrophication.

## Supporting information

S1 Table Effect size for presented models. The effect sizes for linear mixed model analyses of flowering potential for the total community and the exotic, native, forb, C_3_ graminoid, C_4_ graminoid, and legumes analyses.
(XLSX)
